# Exploring different modelling approaches to forecast the community acute respiratory infections burden in children: an Italian epidemiological time series study

**DOI:** 10.1186/s12889-025-21984-1

**Published:** 2025-02-28

**Authors:** Riccardo Boracchini, Benedetta Canova, Pietro Ferrara, Elisa Barbieri, Pietro Giorgio Lovaglio, Antonio Scamarcia, Giovanni Corrao, Daniele Donà, Carlo Giaquinto, Costanza Di Chiara, Anna Cantarutti

**Affiliations:** 1 Department of Statistics and Quantitative Methods, Division of Biostatistics, Epidemiology and Public Health, Laboratory of Healthcare Research and Pharmacoepidemiology, University of Milan-Bicocca, Via Bicocca Degli Arcimboldi, 8, Milan, 20126 Italy; 2 Department of Medicine and Surgery, University of Milan-Bicocca, Via Cadore 48, Monza, 20900 Italy; 3 Center for Public Health Research, University of Milan-Bicocca, Via Cadore 48, Monza, 20900 Italy; 4 Laboratory of Public Health, IRCCS Istituto Auxologico Italiano, Via Ariosto 13, Milan, 20145 Italy; 5 Department of Women’s and Children’s Health, University of Padova, Via Giustiniani, 3, Padua, 35128 Italy; 6 Department of Statistics and Quantitative Methods and CRISP, University of Milan-Bicocca, Via Bicocca Degli Arcimboldi, 8, Milan, 20126 Italy; 7 Società Servizi Telematici (SoSeTe), Pedianet Project, Via Giacomo Medici 9/A, Padua, 35138 Italy

**Keywords:** Acute Respiratory Infections, Burden, Time series models comparison

## Abstract

**Background:**

Acute respiratory infections (ARIs) in young children pose a significant global health challenge, leading to high rates of illness and death. They are estimated to be the fourth leading cause of mortality worldwide, particularly impacting children under five. This study aimed to identify the most effective time series model(s) for forecasting the epidemiological season burden of ARIs for the current 2023/2024 period in Italy.

**Methods:**

Data on the burden of ARIs’ in children aged 0–14 years were retrieved from Pedianet, an Italian paediatric primary care database which includes over 200 family paediatricians. We analysed monthly incidence rates of ARIs from September 2010 to September 2023, following the typical seasonal pattern of these infections. Several forecasting models were compared to predict the future burden of ARI: Error, Trend, Seasonality (ETS); Seasonal Auto-Regressive Integrated Moving Average (SARIMA); Unobserved Component Model (UCM); and Trigonometric, Box Cox, ARMA errors, Trend, Seasonal (TBATS). We evaluated each model's accuracy by examining the residuals and the Mean Absolute Percentage Error (MAPE). The period between March 2020 and February 2022 was forecasted to represent the normal trend without COVID-19. Model parameters were estimated using the in-sample and out-of-sample approach.

**Results:**

The analysis included data from over 1.4 million cases of ARIs retrieved in children aged 0–14 years. The ETS model was implemented to predict the pandemic period. Overall, our findings suggest that exponential smoothing models as ETS (MAPE = 6.85) and TBATS (MAPE = 6.87) were most effective in predicting future trends in monthly ARIs’ burden compared to other methods (i.e., UCM MAPE = 11.08, and SARIMA MAPE = 25.33).

**Conclusions:**

These findings suggest that exponential smoothing models are preferable for forecasting pediatric ARIs’ burden trends in Italy. However, epidemiological data from the ongoing season are crucial for understanding whether residual pandemic effects continue affecting respiratory infection patterns.

**Supplementary Information:**

The online version contains supplementary material available at 10.1186/s12889-025-21984-1.

## Introduction

Acute Respiratory Infections (ARIs) represent one of the leading causes of morbidity and mortality in the paediatric population, especially in children aged 0 to 5 years, who are more susceptible due to factors such as immature immune systems, close contact with daycare or school settings, and other environmental exposures [1, 2]. The World Health Organization (WHO) has identified ARIs, particularly lower respiratory infections, as a significant global health burden, ranking them as the fourth leading cause of death and disability-adjusted life years (DALYs) [3]. These infections significantly strain healthcare systems, constituting a major reason for paediatric outpatient and inpatient care [4].

ARIs are predictable and seasonal despite generating outbreaks that significantly impact global health and healthcare systems. Understanding the impact of ARIs in real-world scenarios is crucial for effective public health interventions, including vaccination programs and healthcare systems preparedness, to mitigate this public health challenge [5]. Moreover, outlining the epidemiology of ARIs over time, including peaks, trends, and seasonality, can provide valuable insights for health policymakers in implementing targeted surveillance systems and preventing future ARIs outbreaks [6].

Time series models are the widest-used methods to analyze temporal variations in various phenomena. They are particularly well-suited for studying the dynamic nature of infectious disease outbreaks and their fluctuations over time. Some models focus on explaining the natural history of infections in the population, delineating states such as Susceptible, Infected and Recovered (SIR) models, and providing a foundational understanding of transmission and immune dynamics [7]. Others are more oriented to using past values and errors to predict future values, accounting for trends and seasonality (i.e., autoregressive integrated moving average models (ARIMA)) aiding in understanding overall patterns and cyclical variations (Exponential Smoothing and Unobserved Component Models) [8–11]. Lastly, new techniques combine the best-fitting parts of these models to build a new category defined as hybrid models [12].

In the past three years since the COVID-19 pandemic, an increasing number of studies have reported time series approaches to forecasting new cases, hospital admissions and deaths [13, 14]. However, many studies focus on a single type of time series approach, resulting in uncertain accuracy measures regarding forecasting [15]. Assuming that each historical series has inherent characteristics that distinguish them from others; conversely, each family model is able to capture only specific patterns. Testing different methodologies on the same time series is critical to providing the best fit and forecast.

This study conducted a comparative analysis of various time series forecasting methods to determine the most accurate predictor of monthly fluctuations in pediatric acute respiratory illnesses at the community level. The objective was to mitigate the burden on pediatric public health resources during the 2023–2024 season using retrospective data up to 2010 and establish the optimal method for predicting future outbreaks of pediatric acute respiratory illnesses.

## Materials and methods

### Data source

Pedianet (http://www.pedianet.it) is a self-sufficient network of over 200 family paediatricians (FPs) who integrate the Junior Bit software into their clinical practices, forming an established paediatric primary care database. The network encompasses approximately 3% of the Italian paediatric population and was previously comprehensively detailed [16, 17]. Pedianet captures patient-level information, including demographics, health status, clinical symptoms, drug prescriptions, and outpatient diagnoses, which is anonymized and securely stored in a protected cloud environment, identified only by a unique numerical identifier.

### Study design and study population

This epidemiological time series study conducted a comparative analysis of various models to determine the optimal model for explaining the trend and seasonality within the monthly incidence rate of pediatric ARIs. The study cohort consists of all ARIs recorded by the FPs during a 13-year study period spanning September 2010 and September 2023. Each year, the observation period was divided into epidemiological seasons from September 1^st^ to August 31^st^.

### Cohort selection

The study included all children aged 0–14 enrolled in Pedianet between September 2010 and September 2023. Under the Italian National Healthcare Service, FPs are healthcare professionals responsible for conducting routine check-up examinations for preventive and medical purposes at specific stages of the child’s life (i.e. 1^st^ month, 2^nd^-3^rd^ months, 6^th^ month, 8-9^th^ months, 12^th^ month, 18^th^ month, 24^th^ month, 36^th^ month, 5-6^th^ years, 8-10^th^ years, 10-12^th^ years, and 12-14^th^ years of age). To minimize misclassification, children without scheduled well-child visits with their FPs, consistent with previous literature, were excluded [18].

### Definition of exposure

ARIs were identified using the International Classification of Diseases, Ninth Revision, Clinical Modification (ICD-9-CM) codes and a freely-entered text field designed and validated by an expert clinical data manager and divided into Lower Respiratory Tract Infections (LRTIs) and Upper Respiratory Tract Infections (URTIs) (Supplemental Materials, Table S1). All ARI cases from September 2010 to September 2023 were collected. Thereafter, cases were summarized into calendar monthly incidence rates per 1000 person-day.

### Time series models

Several time series models were employed to identify the optimal model for explaining pediatric monthly ARI incidence rate patterns and forecasting future trends. The Seasonal Autoregressive Integrated Moving Average (SARIMA), Error Trend and Seasonality (ETS), Trigonometric seasonality, Box-Cox transformation, ARMA errors and Trend and Seasonal components models (TBATS), and Unobserved Component Models (UCM) models were selected based on their ability to capture different aspects of time series data and forecast accuracy. Each model’s features are described below.

(i) SARIMA is the classic time series model used in several time series studies to capture both non-seasonal and seasonal patterns. It can be summarized as [8]:

1 $$\text{ARIMA }\left(\text{p},\text{d},\text{q}\right)(\text{P},\text{D},\text{Q}{)}_{\text{m}}$$

where *p* is the autoregressive order (AR) necessary to make forecasting based on past values, *d* is the degree of differencing involved, and* q* is the order of the moving average part (MA) to forecast starting from the previous forecast error; while *P*, *D* and *Q* indicate the corresponding seasonal parameters for a *m *seasonal period [8]. The selection of the order of the ARIMA model was based on the Akaike information criterion (AIC) and Maximum Likelihood Estimation (MLE) from *auto.arima ()* function in the package *forecast* [19] using R software. For ARIMA models, a common technique to stabilize the variance in the data (achieve homoscedasticity) is a logarithmic transformation. We applied this transformation to our data before analyzing it with the ARIMA model [20].

(ii) ETS, an innovative state space model for exponential smoothing, is used without a clear trend or seasonal pattern. The core idea behind ETS models is to decompose the time series data into these three components (i.e., error, trend, and seasonality) using exponential smoothing techniques. This allows for more accurate forecasts compared to more straightforward methods like naïve forecasting. Exponential smoothing acts as the foundation for this intricate combination. It allows the model to assign different weights to past observations based on their relevance to the current prediction, giving more weight to recent data points while gradually fading out the influence of older ones. It can be expressed as [20]:

2 $$\text{ETS }(\text{Error},\text{ Trend},\text{ Seasonal})$$

The error component may exhibit an additive (A) or multiplicative nature (M); the trend component can manifest as non-existent (N), additive, or additive with damping characteristics (A_d_); and the seasonal component may be non-existent, additive, or multiplicative. The nature-component-model selection is made on AIC [9, 20]. The analysis was performed in R using the function *ets()* in the package *forecast* [19].

(iii) TBATS are designed to handle intricate seasonal patterns in time series data using a combination of the specified techniques. This approach is particularly effective in capturing dynamic seasonal patterns that may evolve over time. As above, exponential smoothing allows the model to assign different weights to past observations based on their relevance to the current prediction, giving more weight to recent data points while gradually fading out the influence of older ones. Overall, TBATS is a powerful tool for forecasting time series data with complex seasonal behaviour. It’s particularly useful in scenarios where traditional methods may struggle due to non-linearity, diverse seasonality, or evolving trends. The general structure is as follows [20, 21]:

3 $$TBATS (\omega ,p,q,\varphi ,\left\{{m}_{1} ,{k}_{1}\right\},\left\{{m}_{2},{ k}_{2}\right\},\dots ,\left\{{m}_{T}, {k}_{T}\right\})$$

Where *ω* is the smoothing parameter for the Box-Cox transformation to stabilize the variance of the series, *p* and *q* are the parameters of the ARMA process, $\varphi$ is the trend smoothing parameter, and the pairs {*m*_*t*_, *k*_*t*_} identify the seasonality and the corresponding terms of the Fourier series [22]. This model is one of the most versatile, considering nonlinear patterns and autocorrelations in the residuals [20]. Auto model selection is based on the AIC criterion by adding and removing transformations, considering or not the trend and its damping version, with or without ARMA (p, q) residual modelling and using several harmonics to determine the seasonal effects if present. The analysis was performed in R using the function *tbats()* in the package *forecast* [19, 21].

(iv) UCM are powerful tools for decomposing time series data into various underlying components. They combine the adaptability of ARIMA with the interpretability characteristic of smoothing models to capture the complexity of temporal variations through the decomposition into clearly defined components. Its terminology per se highlights the focus on capturing the underlying "structure" of the data rather than just fitting a model to the observed values. They assume that the observed data combines several unobserved components, hence the "unobserved" part of the name. Overall, UCMs are valuable tools for time series analysis when the goal is to gain insights into the underlying structure and driving forces behind the data. They excel at decomposing complex patterns into interpretable components, aiding in understanding past trends and making informed predictions. They can be expressed as [22]:

4 $${Y}_{t}={\mu }_{t}+{\gamma }_{t}+{\sigma }_{t}+{\varepsilon }_{t}$$

With *Y* the observed series, $\mu$ the trend to capture the overall direction, $\gamma$ the cycle to identify periodic variations, $\sigma$, the seasonal component reflects regular and repetitive variations at specific intervals, ε, and the residual error represents fluctuations not explained by the previous combine factors [22]. The model was selected by adding/removing/modifying the components and comparing them with the AIC. The analyses were performed using PROC UCM in SAS software version 9.4 (SAS Institute, Inc., Cary, NC, USA).

### Forecast assessment

The historical series was divided into in-sample (60%) and out-of-sample (40%) data for parameter estimation and model validation. The parameters were estimated on the training set, and the selected models were performed on the test set. The more efficient model was used to predict the whole series.

To compare the accuracy of the four models in predicting future values, we calculated the Mean Absolute Percentage Error (MAPE) metric as:

$$MAPE=\frac{1}{N}{\sum }_{t =1}^{N}\left|\frac{{y}_{t}-\widehat{{y}_{t}}}{{y}_{t}}\right|*100$$

where *y* is the observed and $\widehat{y}$ the fitted series from periods t = 1 to N (total number of observations). The metric is independent of scale since it relies on percentage errors, making it unit-free. Using a percentage measure of accuracy is always preferable when comparing forecasts on different time series [23]. Some disadvantages are infinite or undefined results with zero data points and a bias towards negative errors [24].

### Statistical analysis

The socio-demographic and clinical characteristics of the cohort were described through frequencies and percentages for categorical variables and median and interquartile range (IQR) for continuous ones. Descriptive statistics were used to check if the data aligns with the existing literature.

The time series was examined by applying the Seasonal and Trend decomposition using the LOESS (STL) method. This robust approach employs locally fitted regression models with LOESS smoothing to decompose the time series into trend, seasonal, and remainder components. The Augmented Dickey-Fuller and the Seasonal Kendall Test assessed stationarity and monotonic trends, respectively.

We used a counterfactual approach to model the hypothetical epidemiological situation in Italy from March 2020 to February 2022, assuming no COVID-19 outbreak, constructing a model that represents the “normal” trends would have been without the pandemic.

For each of the four-time series models previously mentioned, (i) first, we predicted the months heavily impacted by the COVID-19 outbreak (March 2020 to February 2022) using data on previous epidemiological seasons considering in-sample (September 2010—February 2016) and out-of-sample (March 2016—February 2020) period; and (ii) second, we forecasted the upcoming epidemiological season (October 2023 to September 2024) based on data spanning September 2010 to September 2023 (in-sample: September 2010—August 2018 and out-of-sample: September 2018 – September 2023), which incorporated the previously forecasted COVID-19 period (March 2020 to February 2022).

The underlying assumptions of each model were evaluated through the model's residuals to assess whether (i) the spread of the residuals is consistent (no heteroscedasticity), (ii) the residuals are normally distributed, and (iii) there is any correlation between the residuals at different points in time. The final performance of the different proposed approaches was compared using an appropriate error measure, the MAPE.

A < 0.01 two-sided *p*-value was considered significant for all the tested hypotheses. The statistical analyses were performed using SAS software version 9.4 (SAS Institute, Inc., Cary, NC, USA) and R Statistical Software version 3.6.1 (R Foundation for Statistical Computing, Vienna, Austria). We followed the strengthening of the reporting of observational studies (STROBE).

## Results

Over two-thirds of children (236,642 / 345,877) included in the study from September 2010 to September 2023 had at least one ARI diagnosis. Of the 236,642 children, they reported a total of 1.4 million ARI cases. Among them, 769,637 (53.19%) were found in males, with higher prevalence in children aged 0 – 2 (34.09%) and 5 – 11 (35.75%) years at infection. LRTIs were less frequent compared to URTIs (16.75% vs 83.25%, respectively), also presenting a lower median age (3 vs 4 years, respectively) (Table S2).

Figure S1 displays the monthly incidence rate time series of ARIs spanning the study period from September 2010 to September 2023, along with the STL decomposition. Notably, the Augmented Dickey-Fuller test confirms the stationary nature of the time series (*p* = 0.01). A consistent trend is shown throughout the entire timeframe, featuring a gradual decline in the incidence rate over the years (*p* < 0.01). The time series revealed distinct seasonal variation, characterized by fluctuations and identifiable peaks in incidence during the winter to early spring months. In the remainder component, outliers emerge in the COVID-19 years, indicating a pronounced decrease in the incidence rate during this period.

Tables 1 shows the chosen models and their referred accuracy in terms of MAPE according to the two different forecast periods: (i) the COVID-19-related period and (ii) the overall period and the forecast assessment method.

**Table 1:** MAPEs for in-sample and out-of-sample approach in the two different forecast period

Model	In-sample	Out-of-sample	Overall
A) September 2010—February 2020			
ETS(M,N,M)	6.93	5.43	6.65
TBATS(0.485,{0,0},-,{< 12,5 >}	7.11	5.61	7.77
ARIMA(1,0,0)(1,1,0) [12] with drift^a^	14.60	19.98	17.62
UCM with irregular, level, slope and seasonality modelled with a fourth degree spline and 6 knots from December to May	12.27	10.40	10.97
B) September 2010 – September 2023 with forecasted COVID-19			
ETS(M,N,M)	6.69	7.46	6.85
TBATS(0.123,{0,0},-,{< 12,5 >}	6.72	6.81	6.87
ARIMA(1,0,0)(2,1,0) [12] with drift^a^	13.07	29.94	25.33
UCM with irregular, level, slope and seasonality modelled with a fourth degree spline and 6 knots from December to May	11.70	10.20	11.08

^a^Applied on log-transformed series

**Fig. 1:**
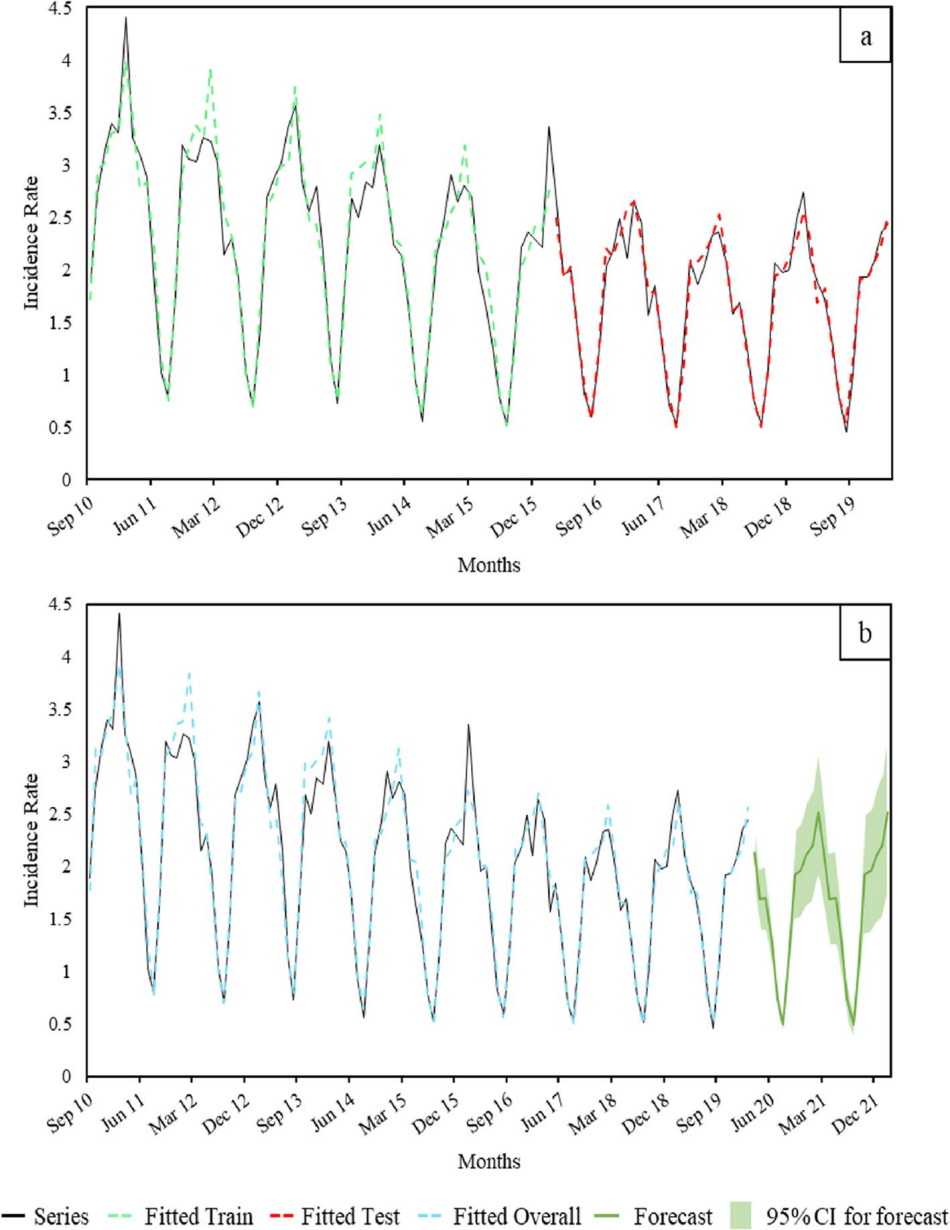
ETS forecast assessment (**a**) and estimation (**b**) of the ARI monthly-IRx1000 person-day, seasons 2010 – 2020. Series contains original data; Fitted Train and Fitted Test are referred to the parameters estimation and validation, respectively; Fitted overall are the results of the validated models application; Forecast and the correspondent 95% confidence interval explain the pandemic years prevision (March 2020 – February 2022). Abbreviations: ETS, Error, Trend and Seasonality; ARI, Acute Respiratory Infection; IR, Incidence Rate

The best models for fitting the in-sample period in forecasting the months impacted by the COVID-19 outbreak (i) were a SARIMA with drift, p = P = D = 1 and the others parameters equal to zero; an ETS with multiplicative error, none trend and multiplicative seasonality; a TBATS with a Box-Cox transformation based on value 0.485, no ARMA error, no trend, monthly seasonality and 5 terms of Fourier; and a UCM with irregular, level and slope component and a spline with fourth degree and 6 knots from position four to nine to model the seasonal component. The ETS has produced a lower MAPE (6.93), similar to the TBATS (7.11), compared with the SARIMA (14.60) and the UCM (12.27). By validating the models in the out-of-sample set of data (March 2016 – February 2020), all the models exhibited a lower MAPE except the SARIMA (19.98 vs 14.60, respectively, for in- and out-of-sample). As the ETS maintained high performance in the test set (MAPE = 5.43), this model was used to forecast COVID-19 years with an overall MAPE of 6.65 (Table 1A, Fig. 1).

**Fig. 2:**
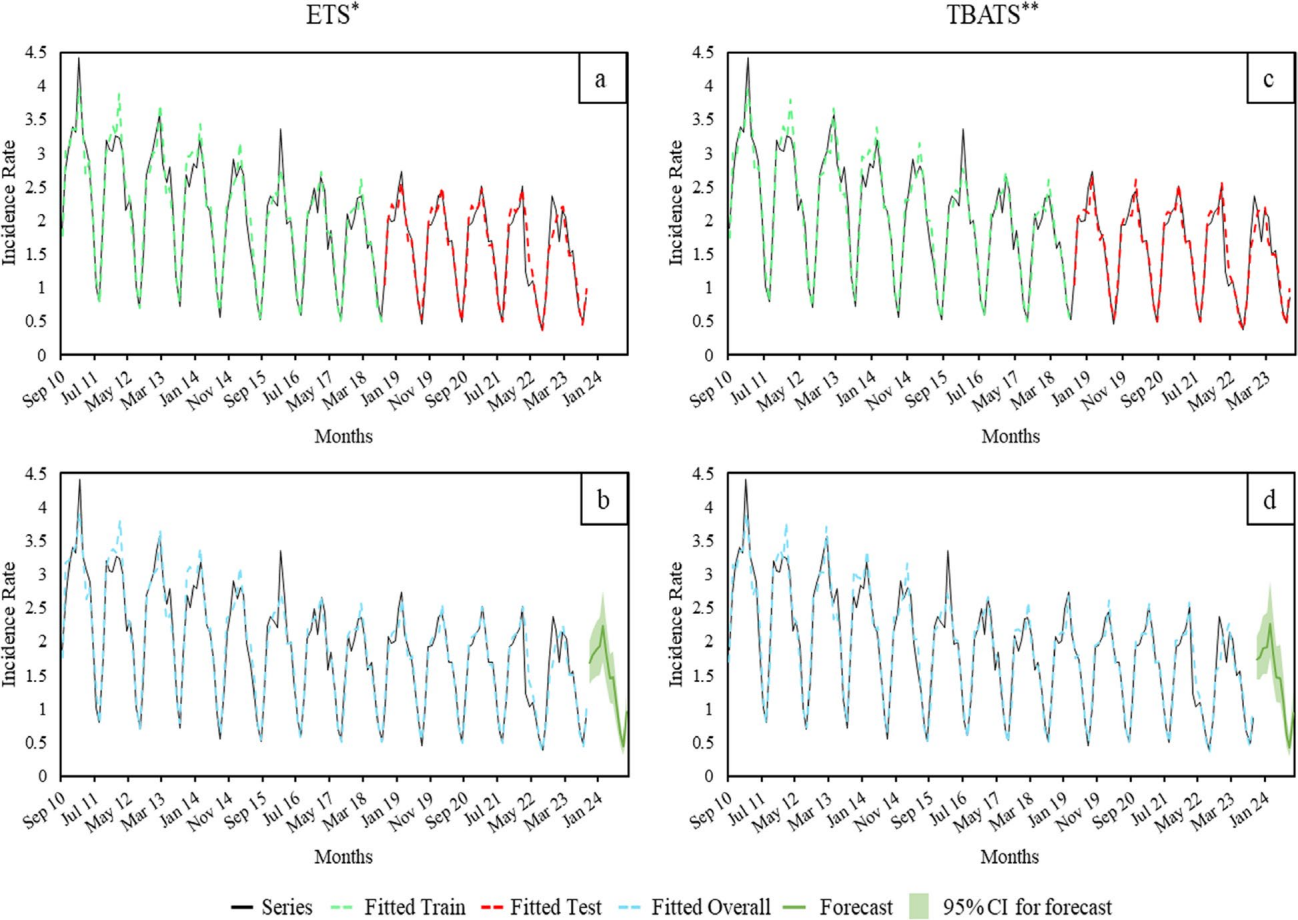
Forecast assessment (**a**-**c**) and estimation (**b**-**d**) of the ARI monthly-IRx1000 person-day, seasons 2010–2023. *, Error Trend and Seasonality; **, Trigonometric seasonality, Box-Cox transformation, ARMA errors and Trend and Seasonal components; Series contains original data with the pandemic years forecasted with an ETS model; Fitted Train and Fitted Test are referred to the parameters estimation and validation, respectively; Fitted overall are the results of the validated models application; Forecast and the correspondent 95% confidence interval explain the current epidemiological season prevision. Abbreviations: ARI, Acute Respiratory Infection; IR, Incidence Rate

Furthermore, the best models for fitting the whole series (i.e., from September 2010 and September 2023) in forecasting the upcoming epidemiological season (ii) were characterized in the in-sample by a SARIMA with drift, *p* = D = 1, *P* = 2, and the others parameters equal to zero; an ETS with multiplicative error, no trend and multiplicative seasonality; a TBATS with a Box-Cox transformation based on value 0.123, no ARMA error, no trend, monthly seasonality and five terms of Fourier; a UCM with irregular, level, fixed slope and a fourth degree spline with six knots from December to May component. Similar to the previous forecast, this prediction again identifies the ETS and TBATS models as the most accurate, with MAPE values of 6.69 and 6.72, respectively. The UCM and SARIMA models follow, with MAPEs of 11.70 and 13.07, respectively. In the validation phase, the TBATS showed a lower MAPE (6.81), while in the overall period, no differences were found in using the TBATS or the ETS (MAPE 6.87 vs 6.85, respectively) (Table 1B, Fig. 2).

By evaluating the parameter selection disparities for the two forecasts utilizing the same modelling framework, we can see that the ETS and the UCM maintained the same structure, the TBATS decreased the Box-Cox transformation parameter, and the SARIMA varied in the autoregressive order.

## Discussion

Different time series models were compared to predict the pediatric monthly incidence rate of acute respiratory illnesses (ARIs) in Italy. Initially, we predicted the period from March 2020 to February 2022, hypothesizing the scenario without the COVID-19 outbreak. The ETS (M,N,M) model outperformed others, with a MAPEs of 5.43. Subsequentially, we focused on predicting ARIs for the ongoing 2023/2024 season, factoring in the simulated COVID-19 period. Both TBATS and ETS (M,N,M) emerged again as the most accurate models (MAPE 6.81 and 7.46, respectively), followed by UCM and SARIMA (MAPE 10.20 and 29.94, respectively). The in-sample and out-of-sample approach was adopted to ensure prediction appropriateness.

We selected models from two common forecasting families, ARIMA and Exponential Smoothing (ETS, TBATS, UCM), covering a wide range of forecasting tasks. While some Exponential Smoothing models (linear ones) can be represented by ARIMA models, the more complex non-linear Exponential Smoothing models lack direct ARIMA equivalents. Conversely, there are ARIMA models that cannot be replicated using Exponential Smoothing [20]. In order to achieve homoscedasticity before ARIMA implementation, a logarithmic transformation has been applied.

As we have proposed a model comparison study, using a monthly time series was an acceptable balance between the prediction’s accuracy and the model’s adaptability to the data. However, if the intention was to do surveillance with the need to make short-term predictions, a weekly time series would be recommended to guide healthcare providers in targeted and prompt interventions.

Our data were representative of the epidemiological burden associated with ARI. We observed that males are more susceptible to contracting ARI, as confirmed by Hasan et al. with 2.9% vs 2.5% and Chobe et al. with 20.5% vs 7.2% of males and females with confirmed ARIs, respectively. [25, 26] Furthermore, consistent with previous literature, children under five years of age were found to be more vulnerable to suffering from ARIs [1, 2].

Our evaluation using metrics like MAPE revealed that the SARIMA model had higher error rates than models like ETS and TBATS when fitted to the ARIs’ monthly time series. This suggests that SARIMA may not be able to fully capture the intricate patterns within this specific data set. This aligns with previous studies on infectious diseases. Wang et al. and Kuan et al. showed that ETS performs better than ARIMA models. [27, 28] In our case, the TBATS and ETS models, which inherently use exponential smoothing techniques, likely excel due to their capability to handle such complexities. This allows for more accurate forecasts compared to more straightforward methods like naive forecasting (e.g., simple average, last value, moving average methods). Exponential smoothing acts as the foundation for this intricate combination. It enables the model to assign different weights to past observations based on their relevance to the current prediction, giving more weight to recent data points while gradually fading out the influence of older ones. However, TBATS has a more complex structure, adding Fourier’s term to model seasonality, allowing forecasting more effectively with the anomalies in the last year. Furthermore, the reason why exponential smoothing models outperformed the others can be seen in the decomposition in Fig. 1. The consistent and stable seasonal pattern over time observed in our data drive this superior performance, as the model’s ability to effectively capture these patterns enhances its predictive accuracy. Sorokina et al. demonstrated the superiority of TBATS vs SARIMA in predicting severe acute respiratory infections. [29] While the UCM model performed reasonably well in our comparison, it has not received much attention in previous research and lacks essential features for directly modelling ARIs at the community level. Interestingly, all our decomposition-based models concurred in excluding the trend component, suggesting its stability over time and reinforcing the consistency of our model selection process.

A major strength of our study lies in the nature of the data. There is no evidence in the literature of research that analysed community-based ARIs in such a large population of almost three hundred thousand children with more than a million infections. As the data came from the FP ambulatorial practice, the representativeness of the ARI’s burden is more accurate with respect to using data from different healthcare sources. This allowed us to train our models in a representative framework to guide future epidemiological burden predictions. Our monthly forecasting of ARI outbreaks is a crucial tool for the public health service to minimize the epidemiological burden of these illnesses and ensure a more efficient response to seasonal fluctuations. This enables them to optimize staffing, medication stockpiles, and other resources to manage the influx of patients effectively. Furthermore, compared to machine learning models, the tested models are easy to implement, still achieving more than satisfactory results.

Our study has potential limitations. First, emerging complex hybrid and machine learning models were developed to study time series data, potentially contributing to improving forecast accuracy. Additionally, the epidemiological season 2022–2023 may still present anomalous characteristics due to the COVID-19 pandemic, biasing new ARI burden estimates. Third, a misclassification is possible in the case in which the FPs do not report the ARI case. Moreover, our predictions are derived from forecasts made during the COVID-19 years. Nevertheless, these years do not influence model selection as they are not included in the in-sample data. Furthermore, the predictions for the new epidemiological season assign greater weight to recent observations (i.e., 18 months after the predicted years).

## Conclusion

Although our models showed promising results, incorporating data from the ongoing epidemiological season remains crucial for accurately defining ARI trajectories in the post-COVID-19 era and guiding targeted interventions. Given the inherent variability of ARIs, we reiterate the recommendation of employing multiple time series models to identify the best fit for specific prediction tasks. Furthermore, the user-friendliness and adaptability of the presented models make them valuable tools for researchers and practitioners alike. Additionally, this study highlights the importance of ongoing monitoring and exploring new models to improve our understanding and prediction of pediatric ARI trends, representing a promising strategy for preparedness programs. The clinical implications of these findings should be further explored through larger multicenter studies, encompassing countries from various hemisphere, to better predict the ARIs epidemiology and promptly inform public health entities.

## Supplementary Information

Supplementary Material 1.

## Data Availability

Data may be obtained from a third part and are not publicly available.

## Abbreviations

ARI: Acute Respiratory Infection
WHO: World Health Organization
DALY: Disability Adjusted Life years
SIR: Susceptible Infected and Recovered
ARIMA: Autoregressive Integrated Moving Average
FP: Family Paediatricians
ICD-9-CM: International Classification of Diseases Ninth Revision Clinical Modification
LRTI: Lower Respiratory Tract Infection
URTI: Upper Respiratory Tract Infection
SARIMA: Seasonal Autoregressive Integrated Moving Average
ETS: Error Trend and Seasonality
TBATS: Trigonometric seasonality Box-Cox transformation ARMA errors and Trend and Seasonal components
UCM: Unobserved Component Models
AIC: Akaike Information Criterion
MLE: Maximum Likelihood Estimation
MAPE: Mean Absolute Percentage Error
IQR: Interquartile Range
STL: Seasonal and Trend decomposition using the Loess
STROBE: Strengthening of the Reporting of Observational studies
IR: Incidence Rate
