# Supplementary material for: Exploring different modelling approaches to forecast the community acute respiratory infections burden in children: an Italian epidemiological time series study
Source: BMC Public Health. 2025 Feb 28;25:810. doi: 10.1186/s12889-025-21984-1 (PMC11869704; doi:10.1186/s12889-025-21984-1)
Supplement: Supplementary file 1 — Supplementary Material 1. [file 12889_2025_21984_MOESM1_ESM.pdf]

Exploring Different Modelling Approaches to Forecast the Community Acute Respiratory Infections Burden in Children: An Italian Epidemiological Time Series Study

Correspondence: riccardo.boracchini@unimib.it, +39 3453242013 (phone number)

Additional file 1

Table S1. Acute Respiratory Infections classifications accordin to the International Classification of Diseases, Ninth Revision, Clinical Modification (ICD-9-CM).

Figure S1. ARI's monthly time series expressed in 1000 person-time with STL method.

Table S2. Sociodemographic and clinical characteristics of Acute Respiratory Infections.

Table S1. Acute Respiratory Infections classifications according to the International Classification of Diseases, Ninth Revision, Clinical Modification (ICD-9-CM).

**Diagnosis**	**ICD-9-CM code**
Lower Respiratory Tract Infections	
Pneumonia	481 Pneumococcal pneumonia [Streptococcus pneumoniae pneumonia]<br>482 Other bacterial pneumonia<br>483 Pneumonia due to other specified organism<br>484 Pneumonia in infectious diseases classified elsewhere<br>485 Bronchopneumonia, organism unspecified<br>486 Pneumonia, organism unspecified
Other	466 Bronchiolitis<br>490 Acute bronchitis<br>491 Chronic bronchitis<br>519.11, 786.07 Wheezing episode
Upper Respiratory Tract Infections	
Pharyngitis	034 Streptococcal sore throat and scarlet fever<br>462 Acute pharyngitis<br>463 Acute tonsillitis
Sinusitis	461 Acute sinusitis<br>473 Chronic sinusitis
Suppurative otitis media	382 Suppurative and unspecified otitis media
Non-suppurative otitis media	381 Non-suppurative otitis media and Eustachian tube disorders
Other	464 Laryngitis – tracheitis<br>460.x Upper respiratory tract infection

Figure S1. ARI's monthly time series expressed in 1000 person-time with STL method.

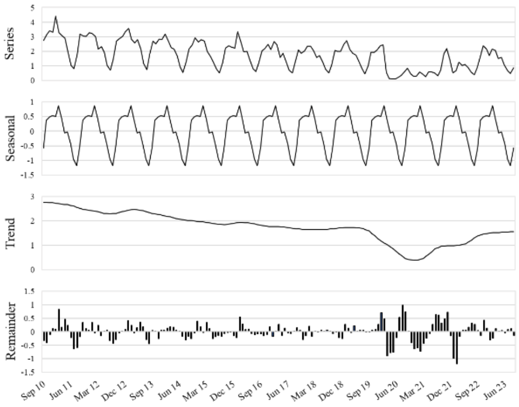

Abbreviations: ARI, Acute Respiratory Infection; STL, Seasonal and Trend decomposition using the Loess.

Table S2. Sociodemographic and clinical characteristics of Acute Respiratory Infections

	**Overall**<br>N = 1 447 033	**LRTIs**<br>N = 242 412	**URTIs**<br>N = 1 204 621
Sex, N(%)			
Male	769 637 (53.19)	133 953 (55.26)	635 684 (52.77)
Female	677 396 (46.81)	118 459 (44.74)	568 937 (47.23)
Age at ARI, median (q1 – q3)	4 (2 – 6)	3 (2 – 6)	4 (2 – 6)
0 – 2 years	493 336 (34.09)	88 912 (35.85)	406 424 (33.74)
3 – 4 years	369 035 (25.50)	68 543 (28.28)	300 492 (24.94)
5 – 11 years	517 283 (35.75)	78 615 (32.43)	438 668 (36.42)
12 – 14 years	67 379 (4.66)	8342 (3.44)	59 037 (4.90)

Abbreviations: LRTI, Lower Respiratory Tract Infections; URTI, Upper Respiratory Tract Infections; ARI, Acute Respiratory Infection.
